# Oophorectomy for Cancer of the Breast

**Published:** 1900-10-27

**Authors:** 


### OOPHORECTOMY FOR CANCER OF THE BREAST.

One of the most remarkable facts in surgery—if it be a fact—and one which is entirely unexplained, however many may be the hypotheses put forward in regard to it, is the effect of oophorectomy upon cancer of the breast.

There are many difficulties in the way of coming to any definite conclusion as to the utility of this proceeding, the great and standing one being that, in the present state of our knowledge, and in view of the probability of being able to give long, if not permanent, relief by removing the breast and its cancer altogether whenever it is seen in an early stage, no one would feel justified in undertaking a purely speculative operation like oophorectomy in early and therefore hopeful cases. Hence, it is only in the inoperatable and otherwise hopeless cases that this operation can be done, and one need not, therefore, be surprised that the results shown are not altogether encouraging. But it must be remarked that the extraordinary improvement in some cases, and the fact that any improvement at all should be found to occur in others, are sufficiently surprising, and go far to encourage a hope, not exactly that oophorectomy may be found to cure, but that its success may give the clue to the direction in which cure is to be sought. Mr. Stanley Boyd has recently reviewed the whole subject and collected certain details concerning forty-one cases in which this operation has been done by various surgeons, thirteen of these having been operated on by Mr. Stanley Boyd himself, and one by Mrs. Boyd. Summing up the matter, he says that oophorectomy should be offered in cases, other than the very acute, in women over forty, with no visceral or bony lesions, in fair condition, and before the menopause. He adds that the work must still be experimental, and that while the surgeon must decide what it is right to do in each case, the patient or the patient's friends should understand the experimental nature of the treatment. He would combine the oophorectomy with removal of the breast in cases in which the disease was in such an advanced form that he could not hope for prolonged immunity from a local operation. It should be noted that in the hands of several surgeons the administration of thyroid extract has been combined with the operation.

